# Meaning-Making of Motherhood Among Mothers With Substance Abuse Problems

**DOI:** 10.3389/fpsyg.2021.679586

**Published:** 2021-09-08

**Authors:** Siu-ming To, Ming-wai Yan, Cheryl Danielle Lau

**Affiliations:** Department of Social Work, The Chinese University of Hong Kong, Shatin, Hong Kong, SAR China

**Keywords:** motherhood, substance abuse, parenting, meaning-making, qualitative research

## Abstract

Previous literature has documented the unique challenges encountered by mothers with substance abuse problems, which may hinder the ability to fulfill parenting responsibilities. Since there is evidence suggesting the engagement in meaning-making processes can help individuals reinterpret their transitions into parenthood and cope with parental stress, this study examined the meaning-making processes of motherhood among mothers with substance abuse problems. Sixteen Hong Kong Chinese mothers with a history of substance abuse were purposively selected and invited to narrate their life and maternal experiences in individual interviews. Based on the meaning-making model in the context of stress and coping, whereby global meaning refers to orienting system of an individual and situational meaning refers to the meaning one attributes to a particular situation, the global and situational meanings of participants related to motherhood and substance use, and their reappraised meanings in response to the discrepancies between global and situational meanings were analyzed. Using thematic analysis, the results showed that when faced with an internal conflict between global and situational meanings induced by substance abuse, most participants engaged in the meaning-making process of assimilation. Rather than changing their inherent parental beliefs and values, most participants adjusted their appraisals toward the situation, and hence made changes in their cognitions or behaviors such as making efforts to quit substance use or reprioritizing their parenting responsibilities. The analysis further revealed that being a mother provided a significant source of meaning to the participants in confronting highly stressful mothering experiences induced by substance abuse. Altogether, the findings suggest that a meaning-making approach may have benefits and implications for helping this population reorganize their self-perceptions, gain a clearer sense of future direction in motherhood, and achieve more positive life and parenting outcomes.

## Introduction

Extensive studies have addressed challenges faced by women who have substance abuse problems during pregnancy and motherhood (e.g., Fergusson et al., 2012). Research has mostly focused on the psychosocial and emotional distress of women (Punamäki et al., 2013), their perceived or actual inadequacy in parenting (Brown, 2006; Massey et al., 2012), or the adverse effects of substance abuse on childcare and child development (Barnard and McKeganey, 2004). Simultaneously, there is a growing interest in research that studies the lived experiences and firsthand perspectives of mothers with substance abuse problems. Although most research in this area has been conducted from a third-person perspective, a first-person approach can promote the development of psychological interventions while maintaining ecological validity (Gaj, 2021), which are particularly important for incorporating the personalistic accounts of those with substance abuse histories. Among available qualitative studies examining personal accounts of motherhood and substance abuse (e.g., Virokannas, 2011; Silva et al., 2012; Torchalla et al., 2014), there is little research examining the meaning-making process in the context of stressful mothering experiences related to substance abuse.

Meaning can be understood as the significance individuals assign to different events or areas of their lives, and is a self-constructed, cognitive orienting system influencing the sense of life purpose, interpretation of lived experiences, feelings of fulfillment, and behaviors (Wong, 1998; To, 2015) of an individual. Likewise, meaning-making can include the restoration of meaning in the context of highly stressful situations (Park, 2010; To et al., 2018). Because meaning-making can facilitate personal growth and provide new life and maternal perspectives, it is possible that enhancing the meaning-making processes of mothers with substance abuse problems can support these individuals in reinterpreting their transitions into motherhood and in coping with maternal stress induced by substance abuse (Tedeschi and Calhoun, 1995; Sawyer and Ayers, 2009). For instance, studies have found that becoming a parent is linked to increased motivation for stopping substance use (e.g., Crozier et al., 2009; Radcliffe, 2011). Other studies have shown that mothers with substance abuse problems tend to struggle with their perception of motherhood, which affects the fulfillment of their parental roles (e.g., Virokannas, 2011; Silva et al., 2012). In contrast, if mothers with substance abuse problems can reinterpret personal challenges and make meaning from stressful life events, it is possible that the women can overcome struggles and carry out their mothering responsibilities.

Moreover, the meaning of motherhood and the identification with being a mother may become increasingly salient during pregnancy or after childbirth as the mother becomes tasked with providing for the needs of her child, tending to other maternal responsibilities, and changing her perception of herself in relation to others, including to her child (Smith, 1999). Some studies suggest that mothers with substance abuse problems often regard their child as the most important person in their life and as a primary source of meaning in their lives (Brownstein-Evans, 2001). Such findings may suggest that the meaning derived from motherhood may help mothers struggling with substance abuse to combat the label of being a “bad mother” by allowing them to reconsider their views on fulfilling the needs of their children or to change their behaviors and practices to be more available for their children (Brown, 2006).

For example, mothers who engage with or enhance their existential wellbeing tend to report fewer experiences of substance abuse and lower levels of parenting stress (Lamis et al., 2014). Additionally, women who discontinue substance abuse during pregnancy are found to report greater feelings of self-worth and decreased symptoms of anxiety and depression (Massey et al., 2012). Taken together, there may be a relationship between the meaning derived from being a mother and the ability of a mother to reconstruct her personal and maternal identities, improve parenting and parent–child relationships, and enhance motivation to change her substance abuse behavior. However, because prior studies concerning mothers with substance abuse problems are focused on the results of meaning-making of motherhood, the psychological mechanisms underlying such meaning-making processes remain unclear.

Overall, exploring meaning-making from the perspective of mothers with substance abuse problems is still relatively understudied. Given the unique challenges and circumstances often faced by these women, it is important to develop a deeper understanding of the interplay between motherhood and substance abuse, as well as the complex meaning-making processes underlying these interactions to provide better support and services to these individuals. Furthermore, meaning is not only personally created, but is also interpersonally constructed and broadly constituted by the ideologies of the wider socio-cultural environment, whereby the differing experiences of socialization may result in differences in the cognitions or sense of identity of an individual (Ugazio and Castiglioni, 1998; To, 2015).

In the process of meaning-making, negative self-perceptions derived from social comparisons and societal stigma may cause mothers to negate their own lived experiences and devalue their personal lessons and learnings (Brownstein-Evans, 2001). For mothers with substance abuse problems, the internal conflict between wanting to be seen as a “good mother” and being identified as a “bad mother” because of their substance abuse may lead to feelings of parental guilt and dysfunctional parenting practices (Baker and Carson, 1999; Brown, 2006; Silva et al., 2012). Furthermore, perceptions of societal disapproval toward mothers with substance abuse problems may interfere with their ability to draw meaning from their own experiences. Consequently, there is a need to understand how mothers with substance abuse problems can cope with social comparisons and societal stigma through creating meaning from their motherhood experiences.

In Hong Kong, the dominant cultural conceptions of motherhood tend to hold mothers morally responsible for the outcomes of their children. For instance, a previous qualitative study reported that Chinese mothers often blamed themselves for the perceived “imperfections” of their children (Pun et al., 2004). Another study found that in comparison with fathers, Hong Kong mothers are usually expected to take more responsibility in childrearing activities (To, 2015). As a result, these mothers were found to suffer from a higher level of parental stress and a lower level of parental satisfaction compared to the fathers. Likewise, another prior study found that single mothers from divorced or poor families are more likely to regard themselves as inadequate mother due to their perceived inability to contribute to a stable future for their children (Leung, 2016). Taken together, these studies demonstrate how Hong Kong Chinese mothers may have manifested the surrounding cultural discourses in their personal construction of maternal meanings. For example, in contemporary Hong Kong society, there are said to be expectations for mothers to be highly involved in the lives of their children, and there is also a mother-blaming culture (Shek and Sun, 2014), both of which may adversely affect the meaning-making and personal growth processes of mothers.

### Analytical Framework

**Figure 1:**
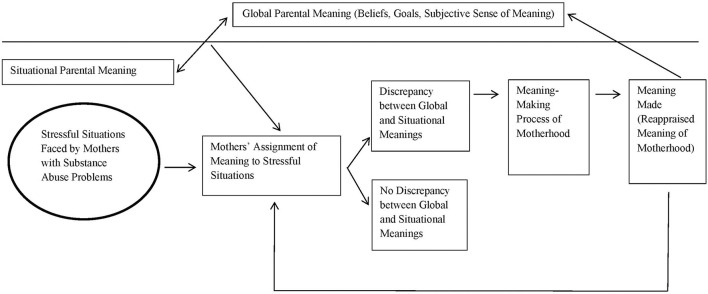
Analytical framework based on Park's (2010) meaning-making model in the context of stress and coping.

The meaning-making model in the context of stress and coping (Park, 2010) was used to inform the analytical framework of this study (Figure 1). According to Park and Folkman (1997), while there are different theoretical perspectives on meaning-making processes in the context of stress and coping, most of such theories examine the functions of meaning-making in relation to how people appraise and cope with stressful events and circumstances. Among the theoretical perspectives, a few common tenets regarding the global meaning and situational meaning have been identified (Park, 2010). First, global meaning involves using the orienting systems of an individual to provide the cognitive frameworks to assist with interpreting life experiences and setting life goals (Pargament, 1997). Individuals who feel their lives are aligned with such orienting systems tend to feel more fulfillment and purpose in their lives (To, 2015). Second, situational meaning refers to the meaning associated with a particular context or event (Park and Folkman, 1997). When stressful life events challenge such global meaning systems, people may encounter discrepancies between their global and situational meanings and thus, experience psychological distress.

In the context of motherhood, global parental meaning can be described as the parental values and beliefs informing the goals of mothers and meanings related to motherhood, while situational parental meaning is related to the interaction between the global meaning of a mother and a surrounding event or circumstance (To et al., 2018). Given that substance abuse problems are generally described to violate core global beliefs about motherhood due to the societal stigma and judgment against maternal substance abuse (Stegnel, 2014; Stone, 2015), this may influence the extent to which a mother perceives her substance abuse problems as inconsistent from her global beliefs regarding motherhood.

To create meaning from such discrepancy, the individual reconstructs the appraised meaning of a situation to reduce the discrepancy between the situational meaning and the personal global meaning of the individual. This is a process referred to as assimilation (Park, 2010). Conversely, transforming global meaning into one more congruent with the situational meaning is known as accommodation (Park, 2010). Ultimately, when individuals can create meaning through assimilation or accommodation and hence reduce discrepancies between their global and situational meaning systems, they are likely to reduce the sense of internal conflict regarding the stressful event (Park and Folkman, 1997). In the context of motherhood, a mother may change her global parental meaning to meet the needs of the situation (e.g., altering her globally situated parental goals to cater to the needs of her children), or change her situationally appraised meanings to become more congruent with her globally held parental meanings (e.g., perceiving the situational challenge in a more positive light and undergoing internal growth as a parent).

Adopting the meaning-making model to examine the meaning-making processes of motherhood among mothers with substance abuse problems, this analytical framework suggests that a mother may experience psychological distress when perceiving a discrepancy between her global and situational parental meanings, where meanings are influenced by how she perceives stressful situations induced by her substance abuse. When attempting to reconcile discrepancies between her global and situational meanings through meaning-making processes, any resultant changes in her appraisals of global parental meanings or situational parental meanings may constitute as meanings made.

This framework was used to guide the development of qualitative interview guides and the data analysis procedures. In brief, this study explored how mothers with substance abuse problems appraise meaning when adjusting to stressful life situations. It also examined how meaning-making processes may allow mothers who are confronted with stressful life events to reduce their perceived discrepancies between their global meanings and situational meanings, particularly when such stressful events were stemming from their substance abuse problems.

## Materials and Methods

### Study Context

In Hong Kong, an increase in the number of females with substance abuse problems who were of childbearing age was observed throughout the 2000s (Central Registry of Drug Abuse, 2020). However, the specific difficulties faced by mothers with substance abuse problems in combination with the lack of services for these mothers and their children together have raised concerns among both practitioners and the public. Although neither an official definition of mothers involved in problematic substance use nor statistical records of these mothers exist in Hong Kong, there were approximately 975 women that were 21 years or older in Hong Kong who have also reported engaging in substance abuse in 2019. Among this group, 348 were married or cohabitating and 273 were divorced, separated, or widowed (Central Registry of Drug Abuse, 2020).

Various local rehabilitation and supportive services are available in Hong Kong to mothers with substance abuse problems. Moreover, with the support of the Comprehensive Child Development Service (CCDS), organized by the Department of Health and Hospital Authority, and the sponsorship of the Beat Drugs Fund Association, local community-based non-governmental organizations (NGOs) providing substance abuse treatment and rehabilitation services can obtain additional resources to provide counseling and support programs for parents with substance abuse problems. Therefore, research participants were recruited from these community-based NGOs.

### Participants

Purposive sampling is commonly used in qualitative studies where specific settings or cases that can provide rich information are deliberately selected for data collection and analysis (Maxwell, 2012). Using purposive sampling, a sample with varying socio-demographic characteristics and diverse lived experiences and perspectives on motherhood and substance abuse was recruited. Specifically, a total of 16 Hong Kong Chinese mothers were recruited for this study, ranging from 20 to 40 years old. All participants had at least one child who was no more than 6 years of age and presented with a history of psychotropic substance abuse before, after, or both before and after the birth of the child. The participants were receiving casework services provided by two community-based Counseling Centers for Psychotropic Substance Abusers (CCPSAs) in Hong Kong which provide counseling services for people with habitual, occasional, or potential psychotropic substance abuse problems. Such inclusion criteria were imposed in light of research showing that substance abuse is a condition prone to chronic relapse, particularly for mothers with young children, who might feel a strong sense of strain and dissatisfaction with their relationships with partners or their role performance in early motherhood, which can prompt them to relapse (Niccols et al., 2012). Furthermore, mothers with a formal medical diagnosis related to unstable or severe emotional or mental conditions were excluded from participation because the narration of life stories during the intervention may cause further emotional disturbance or mental distress.

As mothers with substance abuse problems have been observed, both locally and overseas, to be a difficult group to reach, the small sample size (*n* = 16) was justified. Moreover, according to guidelines for designing samples for qualitative research (Onwuegbuzie and Leech, 2007), this sample size was sufficient for in-depth, qualitative data analysis. All participants were openly recruited through these two CCPSAs. No potential participants were excluded from this study, and no potential participants had declined participation.

The profile of all 16 participants is shown in Table 1. The majority of participants (*n* = 11) were in their 20s, four were in their 30s, and one was 40 years old. Ten participants had completed junior secondary education, while six had completed senior secondary education. Among the participants, seven were married, three were cohabitated, five were separated or divorced, and one was single. The majority (*n* = 10) had one child, five had two children, and one had three children. Eleven participants reported monthly family income below $20,000 HKD. Most (*n* = 12) reported they had their first experiences with drugs during adolescence or late adolescence. At the time of data collection, nine of them claimed they had abstained from substance use and seven were still partaking in drug use.

**Table 1:** Profile of research participants.

**Pseudonym**	**Age**	**Educational level**	**Marital status**	**Number of children**	**Age of children**	**Work status**	**Religion**	**Monthly family income (HK Dollars)**	**Age of first substance use**	**Substance use within the last 3 months**
1. April	29	Junior Secondary	Married	2	2, 4.5 years	Homemaking	Christianity	$20,001–$30,000	12	No
2. Becky	40	Junior Secondary	Separated	2	3, 18 years	Homemaking	Having no religion	$10,001–$20,000	35	Yes
3. Carol	30	Senior Secondary	Married	2	6 months, 11 years	Homemaking	Christianity	Below $10,000	14	No
4. Doris	22	Junior Secondary	Married	2	6 months, 2 years	Full-time employed	Having no religion	$30,001–$40,000	12	No
5. Eleanor	29	Senior Secondary	Separated	1	4 years	Full-time employed	Having no religion	Below $10,000	19	Yes
6. Flora	29	Junior Secondary	Married	1	2 months	Homemaking	Having no religion	$10,001–$20,000	19	No
7. Gloria	32	Junior Secondary	Divorced	1	2 years	Part-time employed	Having no religion	Below $10,000	17	Yes
8. Heidi	21	Junior Secondary	Cohabitated	1	2 years	Homemaking	Having no religion	Below $10,000	18	Yes
9. Ivy	20	Junior Secondary	Separated	1	1 year	Homemaking	Buddhism	Below $10,000	13	No
10. Judith	26	Senior Secondary	Married	3	1.5, 3, 7 years	Part-time employed	Having no religion	Below $10,000	16	No
11. Katrina	28	Junior Secondary	Married	1	10 months	Part-time employed	Having no religion	$10,001–$20,000	Unknown	No
12. Laura	30	Senior Secondary	Cohabitated	1	1 year	Part-time employed	Christianity	Unknown	13	No
13. Mandy	28	Junior Secondary	Single	2	4 months, 8 years	Homemaking	Having no religion	Unknown	21	No
14. Nicole	30	Junior Secondary	Cohabitated	1	2 years	Unemployed	Buddhism	Below $10,000	20	Yes
15. Olivia	24	Senior Secondary	Separated	1	4 months	Homemaking	Christianity	Below $10,000	13	Yes
16. Polly	27	Senior Secondary	Married	1	11 months	Homemaking	Having no religion	Over $70,000	18	Yes

### Data Collection Procedures

The study was conducted according to the ethical protocol approved by the Survey and Behavioral Research Ethics Committee of The Chinese University of Hong Kong. Permission was also sought from the institution in charge of the counseling centers. A written informed consent of each participant to join this study was obtained before data collection. The study used in-depth interviews for data collection to develop a comprehensive understanding of the lived experiences of mothers with substance abuse.

All interviews were conducted within the two Counseling Centers for Psychotropic Substance Abusers in Hong Kong, where each interview lasted between 1 and 2 h. The interviews were conducted in Cantonese and audio-taped with the written informed consent of each participant. A set of guiding questions was predetermined to develop an interview guide, and these questions were posed during the interview in a more open-ended manner.

Examples of guiding questions include:

- Please describe your substance abuse history and current situation.
- Please describe your experiences and feelings when you found out you were pregnant.
- At the time of pregnancy, what was your personal meaning of being a mother? What were your goals as a mother?
- At present, how would you describe your meaning of motherhood? What are your current goals as a mother after giving birth?
- How would you describe yourself as a mother with a history of substance abuse?
- Please describe your experiences and feelings when it comes to taking care of your child. How do you respond to or manage such feelings?
- How do you cope with the difficulties and challenges in motherhood?
- How do you describe your mother-child relationship?
- How do you interact with other family members involved in the childrearing responsibilities for your child?

### Data Analysis Procedures

Using Chinese word processors, student research assistants first transcribed the entire conversational content of the audiotapes. To ensure anonymity, pseudonyms were assigned to all participants. After checking the accuracy of transcripts, this information was imported into the computer-assisted data analysis software, NVivo 10. The software was used for reading, coding, and identifying emergent themes embedded in the interviews. A seven-phase thematic analysis (Braun and Clarke, 2006), guided by the analytical framework (i.e., the meaning-making model), was then performed to analyze the qualitative data.

The seven phases of the thematic analysis involved the following: (1) Reading and rereading the transcripts to understand the narration of each participant and to identify important raw responses, (2) developing a preliminary coding scheme based on the analytical framework, (3) assigning codes to the narratives of participants by focusing on the meaning-making processes and outcomes related to motherhood, (4) categorizing the codes and identifying broader themes at thematic levels, (5) refining the themes, (6) naming the themes and making sense of the connections between them, and (7) summarizing the major themes and identifying representative quotations that best illustrated each theme.

Several cautionary measures were taken to ensure the trustworthiness of the study. First, following the approach of negotiated agreement (Campbell et al., 2013), an intercoder agreement was established during data analysis. During this process, two researchers randomly selected five transcripts at the initial stage of data analysis and coded them separately based on the preliminary coding scheme. These codes were then cross evaluated, which yielded an intercoder agreement rate of 94.65%. Second, whenever disagreements or discrepancies between understandings of the data occurred, meetings were held to discuss the codes and themes, and to exchange opinions to thoroughly consider possible interpretations of the data. Third, although member checking was not used because the researchers found it difficult for participants to read and give feedback on the transcripts and data analysis products because of their relatively low education levels, field observations were conducted on parenting groups and programs organized by the centers and joined by the research participants. Field notes were also written after each interview and observation. Such triangulation of data provided another way of understanding the meaning-making processes of mothers and mothering behaviors (Maxwell, 2012). Lastly, an audit trail was created by properly maintaining all consent forms, audiotapes, transcripts, procedural notes, and products of data analysis.

## Results

The narratives of the participants revealed that although each mother presenting with substance abuse had previously adopted her own way of making sense of her parental experiences, the recurrence of certain experiences revealed common themes embedded within their meaning-making processes. This section will thus articulate the themes elicited from the narratives of participants, with particular emphasis on the interrelationships between global parental meaning, situational parental meaning, discrepancies between such situational and global meanings, and the meanings made to reduce such discrepancies. The themes and sub-themes of this study are shown in Table 2.

**Table 2:** Themes and subthemes of this qualitative study.

**Component of meaning-making**	**Theme**	**Subtheme**	**Number of participants who addressed the subtheme**
Global parental meaning	Mothers should provide for their children	Mothers should make sure children's physiological needs are met	9
		Mothers should let children grow in an intact family	7
		Mothers should prioritize their children and give them the best	7
		Mothers should be sensitive to the emotional needs of their children	4
	Mothers need to nurture their own children	Mothers are the ones responsible for nurturing their children	7
		Mothers need to be role models and demonstrate good behaviors	6
Situational parental meaning: assignment of meanings to stressful situations	Perceived consequences resulting from internally-attributed situations		10
	Perceived consequences resulting from externally-attributed situations	Issues from being involved with other substance users Issues from relationship conflicts Issues from disadvantaged life circumstances	9 10 14
Discrepancies between Global and Situational Meanings	Negative emotions derived from the discrepancy between global and situational parental meanings	Self-blame Worry Powerlessness	9 8 9
Meanings Made to Reduce the Discrepancies between Global and Situational Meanings	Reconsidering the use of substances		16
	Experiencing personal growth	Re-considering their own career or personal development	11
		Learning to be an author of their own lives	13
	Perceiving their growth as a mother	Striving to be better mothers for their children	15
		Prioritizing their children during life struggles	10
		Striving to provide a healthier environment and lifestyle for their children	14

In the past, I was so full of myself. Substance abuse, hair dyed gold, purple, multi-colored, and smoking. Now, I've even quit smoking. I haven't smoked for 2 years, because I'm worried my son would cough… I had never thought I'd be like this. Even my friends said it's a miracle I could quit drugs, not to say smoking as well. I myself am happy; makes me see my son as my treasure even more… He has motivated me and accompanied me through so many things. (Judith)

In the above narrative, Judith vividly described the drastic changes she made in her way of life as motivated by the birth of her son. In line with the meaning-making model of Park (2010) would predict, when Judith sensed it was impossible to attain her global parental meaning of raising her son while maintaining substance abuse, she was determined to completely change her lifestyle and quit substance use to resolve the discrepancy between her global and situational parental meanings.

Similar to Judith, the mothers with substance abuse problems in the study might not have cared for their own health or life development but instead enjoyed their risk-taking lifestyle before they became mothers. Judging from the substance abuse records of the women, onlookers may expect they would not have functioned well as mothers, nor take full responsibility in childrearing. Contrary to these assumptions, it was found that the stressful situations the women dealt with after childbirth have often become very powerful catalysts to fight hard against their addictive behavior and to reconstruct the meanings of their lives as exemplified by the above narrative from Judith. In this study, the experiences of mothers were investigated to see how parenthood has become the turning point of their lives.

### Global Parental Meaning

In the interviews with the sample of mothers with substance abuse problems, all of the participants mentioned their parental values and beliefs. For instance, all of them discussed the importance of providing for their children, which therefore emerged as the first theme in terms of the global parental meaning of mothers. Regardless of whether they felt they were able to meet the needs of their children, these beliefs appeared to serve as the guiding system they used to appraise themselves and adjust their daily or parenting practices. According to their narratives, it was shown that the participants all held themselves responsible for their children.

Four sub-themes have also been identified. The first sub-theme is that they made an effort to ensure that the physiological needs of their children are met, as indicated by narratives from nine participants. This is reflected in the following excerpt from Doris:

Raising the child, feeding him, and looking after him… Taking care of him even when I am not feeling well is really harsh. But as a mother, I know this is how to be a mother at that moment. (Doris)

A second sub-theme is the mothers felt they should provide their children with an intact family (*n* = 7), illustrated by the following:

Actually, I've always wanted to give my child a family. I also really want a family that belongs to myself. With a father and a mother. I want to give my child a family intact. (Ivy)

Some participants also believe mothers ought to provide the best circumstances for their children (*n* = 7), which is the third sub-theme:

Giving the child the best things. Teaching him the best stuff. (Katrina)

The fourth sub-theme is the mothers believed they need to be sensitive to the emotional needs of their children (*n* = 4), indicated by a quote from Carol below:

Caring about how the child feels. For example, he really wants to share with Mummy about the happy and unhappy events at school. (Carol)

The next prominent theme regarding the global parental meaning of mothers is their belief in nurturing their own children. Within this theme, two sub-themes are identified. First, some specifically pointed out mothers should be the ones responsible for and are suitable for nurturing their own children (*n* = 7). For instance, Katrina expressed:

I don't want my child to be taught by someone else to call me Mum. I'd want to be the one teaching you [her child] to call me Mum, right? (Katrina)

For the second sub-theme, some participants iterated the necessity for mothers to be the role model of their children and demonstrate good behaviors (*n* = 6):

I think the key point is to be a role model. You need to set a good example for the child to follow. (Ivy)

Overall, the participants appeared to believe in the importance of the initiative to cultivate the well-being of their children. The above themes and sub-themes illustrate how these mothers with histories of substance abuse value the well-being of their children, whether it be physiological, developmental, or emotional well-being. Caring for the survival and development of their children appears to be a key component of their general orienting systems as a parent. Although the public may hold preconceived notions or assumptions that mothers with a history of substance abuse are “bad mothers” (Baker and Carson, 1999; Brown, 2006; Silva et al., 2012), these narratives demonstrate how their parental values and beliefs may be similar to mothers without a history of substance abuse.

### Situational Parental Meaning: Assignment of Meanings to Stressful Situations

As a result of their motherhood, the participants had to appraise their life situations as substance users after assuming a new identity as a mother. Their previously tolerated circumstances or behaviors once viewed as acceptable were considered problematic after becoming mothers. The narratives extracted from the interviews reveal entering motherhood magnified previous issues, particularly those stemming from substance use, and introduced new dilemmas into the lives of mothers.

Among the 16 participants, some appeared to have begun their own meaning-making processes and started to make behavioral changes in their lives, such as quitting substance use soon after the confirmation of their pregnancy (*n* = 4). During the interviews, some (*n* = 5) shared they were able to quit substance use entirely after childbirth and reported to have more stable mothering practices. Other mothers (*n* = 7) shared their goal of quitting the use of substances but were struggling to do so due to stressful circumstances.

In the following sections, the narratives of three selected mothers (Flora, Judith, and Nicole) were used to reflect how the participants made meaning from their mothering experiences. These three individuals were selected for each being at a different stage of substance use at the time of data collection, and they also differed in their life circumstances (refer to participants 6, 10, and 14 in Table 1). Thus, the following sections will mainly articulate the common themes elicited from these three narratives, in relation to their assignment of meaning to stressful situations, how they resolved the discrepancies between situational and global meanings, and their meaning-making processes.

The narratives revealed both internally and externally attributed stressful situations faced by the participants of this study, whereby internally attributed situations are those perceived within the control (or lack of control) of an individual while externally attributed situations are those perceived to be affected by factors outside of his or her control.

#### Perceived Consequences Resulting From Internally Attributed Situations

Several participants mentioned their use of substances had caused them to become irritable and feel a lack of control over their emotions or behaviors when caring for their children. For instance, Nicole recalled an incident where she threw her baby off the bed while she was under the influence of substances. She narrated:

There was a time I threw my daughter off the bed very hard… she was about 1 year old… I had taken a drug. She cried desperately. The whole family saw this. This was caused by taking ice [methamphetamine]. (Nicole)

Judith also commented she was often emotionally unstable from her use of substances, which contributed to her reduced tolerance in response to parenting difficulties. Moreover, both Nicole and Judith had previously left their children under the supervision of other family members for an extended period of time due to their substance abuse or the consequences of substance use. This separation with their children had appeared to create greater difficulties in bonding with their children, as illustrated by the narrative by Judith below:

I either looked really mental, or was very unresponsive and still. I could suddenly go crazy… But at that time, I didn't stay at home and didn't like living at home… so I abandoned my daughter for a period of time. I did not see my daughter…for at least 1 or 2 years. My daughter did not like me then. (Judith)

#### Perceived Consequences Resulting From Externally Attributed Situations

Some participants revealed challenges previously faced were often complicated by external factors, which they perceived as out of their control. In light of such narratives, three sub-themes regarding such externally attributed situations were extracted, as highlighted below.

##### Issues from Being Involved with Other Substance Users

The first sub-theme is difficulty stemming from the significant other who also has a history of substance abuse. A common concern raised by the participants is they feel tempted to resume their substance abuse when they are surrounded by others who use substances. Some also expressed worries of potential relationship issues when their significant other was a frequent user of substances. For example, Judith's network of others with substance abuse histories was enlarged when she worked in a night club, where she felt pressure to use substances every day after work with this group of peers. Similarly, in the narrative below, Nicole described her increased substance use during the period when she was involved in an intimate relationship with a partner who uses substances heavily.

Honestly, he [her partner] also took drugs… We took it together, more and more severely… But that day, why did I have to pull him back? I also wanted him [to know] somebody treasures him… I felt very torn. Yes, but I also thought [then], if I am not doing so [to leave her partner], I'm harming myself. (Nicole)

##### Issues from Relationship Conflicts

The second sub-theme emerging from the narratives of participants is centered around the difficulties caused by being in an intimate relationship involving betrayal, abandonment, and violence. The physical and emotional consequences as a result of such relationships were found to complicate the challenges these mothers were already faced with, hence making it more difficult for the mothers to care for their children. Some participants found it especially difficult to overcome issues with a partner who was also the biological father of her child. Judith shared her experiences with violence imposed by the father of the child:

With my husband… We argued a lot. He actually physically strangled me with his hands. What made me heartbroken was when I thought about why did I went through the hardship to bear your [his] baby, and you [he] did this [hit me]? (Judith)

Judith further expressed how she was both physically and emotionally hurt by the violence of her husband but would still choose to endure this because the person involved is the father of her third child, not simply a romantic partner without any connection to her child.

##### Issues from Disadvantaged Life Circumstances

The third sub-theme of external circumstances is related to how mothers faced with life circumstances perceived putting their children in a disadvantaged position, both at the family and community levels.

Because of their own childhood experiences and current situations, many participants perceived themselves having limited capacity to provide adequate care for their children and lacking the appropriate family or household conditions their children would want. For instance, about half of the participants were not engaged in a stable relationship with the father of their children. The narrative of Judith revealed that each of her three children have different biological fathers, which has brought about complications:

I've always thought since young conceiving a baby should, of course, be with the husband. I thought this even during the times when I liked going out to play and go raving… My heart aches when friends ask, “Hey, why do your children have different surnames?” (Judith)

Often, because of an unplanned pregnancy, they felt unprepared when becoming mothers and found it difficult to adjust to sudden changes in their lifestyles and living arrangements. Some mothers felt their neighborhood would pose negative influences on their children. Nicole illustrated how the neighborhood she grew up in, where she used substances, may pose certain social disadvantages to her child:

I also want to rent a better apartment for her to live in, not here [the estate she is living in], where if we go down the street, everybody knows me. Everybody knows me for what I was like. Would I want her [daughter] to know what I was like? I don't want her to know. (Nicole)

### Discrepancies Between Global and Situational Meanings

According to the meaning-making model, appraisals of meaning are influenced by the extent to which an individual perceives certain events violate their values, beliefs, and goals. According to the model of Park (2010), after a person initially assigns meaning to a stressful event, they then determine whether there is a disagreement between the appraised meaning and their global meaning, and the extent of such discrepancy (Park, 2010). Together, this influences the perception of an individual of, or in response to, the event at hand.

In this study, although the participants were found to have constructed situational meanings to better make sense of their life situations as someone with a history of substance abuse, they also continued to hold onto their original global meanings, and in particular, the global meanings positioning mothers as the primary caregiver of her children. Moreover, these mothers shared that their life experiences or current situations as someone with a history of substance abuse were in opposition to their personal values, beliefs, and goals as mothers, which created a sense of discrepancy between their global and situational meanings of motherhood, and thus inflicted negative internal conflicts.

#### Self-Blame

In hindsight, nine participants discussed feelings of regret regarding their past and expressed that they wished they could have provided a better example to their children, especially because they are now aware their past may have consequences for their present status and family. They also described feelings of self-blame and guilt for the potential consequences of their past behaviors may inflict onto present or future of their children. For example, because of the unknown impact of her past substance abuse on the future of her child, Flora described her sense of guilt:

I have gone out and was involved with that type of thing, so perhaps I'm afraid this will in time affect her [child] greatly… If this will later affect her brain development, then I would have really harmed her. I'm really worried. (Flora)

Nicole also blamed herself for having deprived her child of a complete family:

My source of guilt is seeing her not being in a complete family as soon as when she was born having no father. That was already very sad. She even had so many things intubated in her at birth [when she was in hospital]. I wonder if I've harmed her. (Nicole)

#### Worry

Eight mothers were worried their perceived inadequacy to be a positive role model or their perceived inability to provide adequate parenting support would eventually lead their children to copy parent behaviors or parenting in the future.

She might think, “I see Mum was like this before, so why shouldn't I go wild too?” I feel she would learn from what I did in the past and get back at me in that same way… How will I teach her then, how can I ever teach her by role modelling? No matter how much I improve myself, that will not help. (Nicole)

The participants were also worried the problems stemming from their family of origin would be passed down to their children:

In the past, when my mother was not in a good mood, she would say, “Don't talk to me” and then immediately explode and keep scolding… Actually, this could cause other people to have mental illness… So I don't want my kids to be like this when they are older. I am worried things would turn out exactly like that, repeating again [the fate]. (Judith)

#### Powerlessness

As many participants unexpectedly became mothers while they were still using substances, some of them (*n* = 9) described that the sudden need to provide for their children amidst their own stressful life situations had created additional stressors, which complicated their ability to fulfill their new parental responsibilities. For example, Nicole emphasized the importance of taking care of her child on her own, despite having to be away from home for work to financially support her child:

Actually, what I want most is to take care of my child with my own hands, to take care of my daughter, be a good mother… But even though I want to do this, I feel torn. Where do I make money for her?. Only one person, not two, to raise her… Other people have a father and mother. I only raise her by myself. (Nicole)

At the family level, although Nicole knew her partner has a severe history of substance abuse and viewed him as having negative influences on her child, she hesitated to break up with him because she wanted to maintain a whole family structure and provide her child with a father figure:

As we both often took drugs, do you think our tempers would not be hot? I knew we would be in a bad mood and quarreled. But there was no need to let my daughter know why we argued again and again… My struggle was, breaking up with him is not the way, and not breaking up with him is also not the way. I felt trapped in a dilemma. If we broke up, my daughter would lose her father. (Nicole)

### Meanings Made to Reduce the Discrepancies Between Global and Situational Meanings

The meaning-making model suggests that the emotional reactions induced by discrepancies between global and situational parental meanings may prompt one to cope by making meaning to reduce such discrepancies (Park, 2010). For instance, in the present study, the distress caused by the discrepancy between the maternal concerns of mothers and the actual situation at hand appeared to have brought forth a newfound determination to make new meanings to cope with the situation. Based on the narratives of participants, these newly created meanings appear to manifest in three different categories: reconsidering the use of substances; experiencing personal growth; and reflecting on the growth as a mother.

The narratives revealed all participants strived to fulfill their maternal roles and, ultimately, did not abandon their children. This suggests that the mothers opted to preserve their global parental meaning, particularly those in relation to what they believe a “good mother” should be and, instead, adjusted their interpretations toward situational events. Specifically, the mothers demonstrated the use of assimilation during the meaning-making processes. The narratives also showed the mothers used different ways to experience personal growth and enhanced their personal meanings of motherhood to reduce the perceived conflicts between their appraised situational and global parental meanings.

#### Reconsidering the Use of Substances

Flora, Judith, and Nicole have all changed their substance use behaviors at their own pace and to different extents. For example, Flora had quit substance abuse as soon as her pregnancy was confirmed:

The craving is not as strong as before. Before having the baby, I could go out to play. I had to go out, and would not stay at home. In the past, I really couldn't live without it [the drug]. Yes, the difference was quite huge… A big difference. (Flora)

On the other hand, Judith and Nicole shared they sometimes found themselves using substances in a more controlled manner compared to before. For instance, Judith shared she found the strength to quit substance use after the birth of her third child:

I quit because of him [husband] and this son. I haven't been taking drugs for 2 years, I haven't touched it. I'm most healthy with this son. I really won't take it [drugs]. I won't go out, won't go out late at night. I will raise him with my whole heart. (Judith)

Nicole, despite repeated relapses, kept trying to abstain from substance abuse for her daughter:

I still had taken drugs every day, then I had stopped, had taken it again, and had stopped again… Till my daughter said “Mama,” I decided to stop [because] I could not bear this. I did quit, actually for almost a month. I tried hard. I really tried very hard… I still keep trying to force myself to quit it completely. (Nicole)

#### Experiencing Personal Growth

The current findings demonstrate the meaning-making process could lead to personal growth by heightening the awareness of the participants of their life roles and how they can become authors of their own lives. The three sub-themes related to the perceived personal growth of the mothers can be found as follows.

##### Reconsidering Their Career or Personal Development

For instance, Nicole started to work when she realized she had to earn money to provide for her daughter:

I really needed the job. The job, actually was from 6 a.m. till 3 p.m. I could work in the kitchen. I'm okay for morning shifts… If I need to, then I'd work and won't care about anything else. I need to earn money for my daughter… My daughter is my biggest motivation. (Nicole)

Another participant, Eleanor, also shared her return to the workforce after years of not working. Her narratives revealed it was not the need to provide for her child that motivated her to resume work, but rather, she felt she needed to secure her own livelihood. This suggests the mothers may have been concerned with their ability to take care of themselves, in addition to providing for their children. She narrated:

I haven't been working for many years. I've just started to go to work again, so would need to adapt, and really would feel exhausted. But if you say I'm doing this for my daughter…actually, at the beginning, it was for my own livelihood. If I can't even take care of my own livelihood, when she's over 10 years old and studies in primary school, she'd need lots of personal spending. If I only start working then, it'd be too late. (Eleanor)

##### Learning to Be an Author of Their Own Lives

After becoming mothers, several participants shared that despite certain difficulties or perceived limitations, they could still appreciate their capacity for growth and create their own futures. For example,

I like to have fun. But apart from having fun, I've still done my part. I don't need to depend on someone else. Since I can gradually support myself, I don't feel like a failure. (Eleanor)

The mothers appeared to have come to the realization of the significance in what they chose to do with their lives and hence, began to form plans for the future. Particularly, some participants articulated their motivation was not due to the need to comply with external expectations. Rather, they began to plan for the future and reflect on their roles in life:

Now I've quieted down, I think maybe I don't need to be with a man. Maybe I need to think quietly by myself about what I'll be able to do for my daughter. (Nicole)

As the mothers began to focus on the roles that they hold in relational contexts, their relational self also developed. Many mothers not only thought about themselves, but they also took their family members into consideration when contemplating their own future. For example,

If the current living situation is not acceptable, if each day cannot be lived well… Even if my younger brother can take care of my mother when she becomes old, how about my daughter? (Eleanor)

#### Reflecting on the Growth as a Mother

From the analysis of their narratives, it can be uncovered that the participants made efforts to improve their mothering practices and be “better mothers.” Three sub-themes can be further identified among the theme of perceived growth as a mother.

##### Striving to Be Better Mothers for Their Children

Despite their negative self-perceptions, the narratives showed how these mothers continued to make efforts to become “better” mothers, even in face of the challenges caused by their substance abuse. For example, Flora described her attempts to enhance her capacity to care for her children:

There were many things I was afraid I couldn't handle, and there were many things I got to learn, but I was also afraid I was not able to do them. But now I've already changed my point of view. Actually, for many things, you've got to learn them by yourself. Just learn bit by bit and absorb it bit by bit. Because actually, at this moment, you're a blank sheet of paper, since it's your first child. (Flora)

On the other hand, Judith has taught herself to stop throwing a temper to control her child. Instead, she has begun to adjust her parenting approach and expectations:

Now I would immediately try to talk to her [child] softly, “What happened? Mum promises not to scold you anymore and will give you all my love again.” Then she would stop the temper tantrum immediately. So, this method seems to be working. Now, I would know to respond like this. (Judith)

##### Prioritizing Their Children During Life Struggles

Even though the mothers with substance abuse problems reported conflicting priorities, such as catering to their addictions, personal relationships, and tending to their child, many participants were found to prioritize the needs and well-being of their children before their own. This is illustrated by a description of Flora on changing her life focus and prioritizing her child over her own enjoyment:

Everything changed, meaning after my child appeared, my future became different. The road will be walked because of her. I really consider her first for everything. So, she will be prioritized in everything. (Flora)

Furthermore, the narratives demonstrated how some of the mothers were overwhelmed by their personal struggles and yet, still opted to prioritize their children. For example, Judith contemplated suicide to end her emotional turmoil, but was also worried her son would have to suffer as a result:

I have thought of committing suicide, for a period of time… The baby is so cute. He is very fine and wants to grow up. I cannot take his right away from him… I can't take away the right of the child. I would also think, “Hey, if I died, what would happen to my son?” (Judith)

##### Striving to Provide a Healthier Environment and Life for Their Children

Many participants tried to improve their circumstances by providing a positive family atmosphere for their children. This included adjusting their relationship with the father figure, other members of the in-law family, and family of origin. Flora described adjusting her perceptions of her family relationships:

Sometimes, after my in-laws and I are back from work… They go to work, so in the afternoon, after work, they can play with the girl… The relationship among everyone has deepened. (Flora)

Another participant, Polly, mentioned the reason why she had to express gratitude to her mother-in-law:

Need to do something to express gratitude to her [mother-in-law]. If not, my child will be pathetic, because when I go to work I wouldn't know how my mother-in-law would treat him, right? Need to think for my child, not just for myself. (Polly)

Overall, although the participants reported undergoing similar challenges and difficulties due to their substance use, there were variations in the way the mothers appraised the meanings of their situations. This suggests different meaning-making processes may have taken place. Particularly, the discrepancies between the global parental meaning of the mothers and their situational parental meaning may have prompted them to engage in their own meaning-making attempts. In the following discussion, the different meanings made by the participants and the appraisals of their situations in an attempt to reduce discrepancies between their global and situational meanings are expanded.

## Discussion

Previous literature often portrays mothers with substance abuse problems as victims to their situations (Brownstein-Evans, 2001), with emphasis on the challenges faced by these individuals, including mental health problems (Punamäki et al., 2013), social isolation and socioeconomic disadvantages (McClelland and Newell, 2008), family dysfunction and violence (Fare et al., 2008), and other traumatic life experiences (Torchalla et al., 2014). Consistent with literature, the findings of this study also indicate many of the participants had encountered stressful life situations at different levels. However, they had sought to view their pregnancies or their children as the impetus for creating change in their lives, despite facing various difficulties as a result of their substance use (Radcliffe, 2011). The findings of this study indicate that through the process of meaning-making, many participants who initially struggled with their personally and socially created meanings of parenthood were able to reconstruct such parental meanings and hence, experience personal and parental growth. Personal and social meanings are intertwined, and the maternal meaning of an individual is both created on a personal level and constructed based on her surrounding social systems and the socio-cultural environment at large (To, 2015).

Regarding personal meanings, the findings add to the current understanding of personal growth among mothers with substance abuse problems from a meaning-making perspective. These findings appear to be consistent with other studies on personal growth among women following childbirth, which found that changes in the self-perception of the mother toward her life direction are linked to increased resilience, higher levels of maturity, and greater reexamination of life philosophy, and setting of new priorities (Taubman-Ben-Ari et al., 2012; Taubman-Ben-Ari, 2014). Expanding upon the findings of Taubman-Ben-Ari (2014) and Sawyer and Ayers (2009) regarding the outcomes of personal growth, the narratives from this study suggest that the personal growth of a mother not only has a positive impact on her psychosocial functioning, but also serves as an ongoing reflective process regarding her own maternal meanings, life choices, and other parenting challenges. Consequently, both the personal growth resulting from the meaning-making processes of a mother and the meaning-making process itself appear to have positive influences on the life and parenting outcomes of these mothers. In that sense, these findings echo the concept of posttraumatic growth developed by Tedeschi and Calhoun (1995), which perceives that posttraumatic growth is both a positive outcome of effective coping and adaptation, and an evolving process of meaning-making.

Furthermore, the findings indicate the participants who reported less difficulties when quitting substance abuse (*n* = 9) were also the primary caregiver of their children, suggesting these mothers may have connected their substance abuse recovery with their roles as mothers (Gunn and Samuels, 2020). For these participants, their desire to fulfill their role as mothers and their desire to engage in substance abuse created a discrepancy between their global and situational parental meanings, which needed to be resolved. The narratives from this study also revealed most participants opted to reduce such discrepancies through the process of assimilation instead of accommodation, which would have required the mothers to reassign situational meanings rather than to alter their inherent parental values and beliefs.

The narratives showed that all participants have reconsidered their substance use behaviors (*n* = 16). However, some participants (*n* = 7) were still working on changing their substance abuse behaviors and were experiencing difficulties with assigning meaning to stressful situations at the time of data collection, even though these participants still expressed their intention to deliver on their maternal responsibilities. For this group of participants, while substance abuse has caused stressful situations, some have decided to appraise their use of substances as a quick solution to reduce their stress temporarily or to maintain an intimate relationship with their substance-using partners (Virokannas, 2011). The struggles of these mothers were found to be not well-understood or well-accepted by those around them, particularly because others viewed these problems as “self-initiated” and “socially unacceptable” (Baker and Carson, 1999; Virokannas, 2011). Thus, these unresolved substance-related and other relationship issues, compounded with the demands of motherhood (Baker and Carson, 1999), appeared to have caused additional stress to these mothers. Consequently, these mothers may continue to experience negative emotions because of such discrepancies which may, in turn, hinder their ability to create new meanings or form a more positive maternal identity.

With regard to social meanings, the meaning-making of mothers with substance use problems is also said to relate to their daily interactions with others and to their surrounding social contexts (Klee et al., 2002; To et al., 2018). The review of Park (2010) on meaning-making processes in the context of stress and coping has argued that meaning-making should be considered in conjunction with the social constraints inhibiting meaning-making. The findings of this study demonstrate that social environments may also influence how these mothers assign meanings to maternal and child-rearing situations. For example, some participants highlighted that they were originally negatively impacted by their lack of family or social support and infliction of stereotypes from others, which therefore affected their ability to make meaning out of their situations.

In line with previous research examining the family and social environments of mothers with substance abuse problems (Nair et al., 2003), the mothers in this study had to navigate different contextual difficulties such as being involved with a partner who has a substance abuse history (*n* = 9), dysfunctional couple relationships (*n* = 10), and other disadvantaged life circumstances (*n* = 14). All these environmental factors may adversely affect the assignment of meanings to the situations of mothers. For example, their concerns over family relationships and life circumstances may conflict with their parental concerns (Gruber and Taylor, 2006). Although some mothers (like Judith and Nicole) reappraised meanings to reduce the discrepancies between global and situational meanings, they kept the relationships with their partners even in the face of domestic violence and negative influence on substance use. As such, the relational elements of meaning-making were uncovered by the narratives of participants. Specifically, the mothers often referenced their relationships with their partners or family while renegotiating their maternal meanings. As argued by the meaning-making model of Park (2010), positive relational dynamics facilitate the meaning-making process. In this light, how to co-construct parental meanings with family members and partners in the context of motherhood with substance abuse should be thoroughly investigated for the facilitation of the meaning-making of mothers.

Given social, cultural, and gender stereotypes or expectations may negatively affect the self-perception of these mothers, the findings of the study suggest the significance of investigating how mothers with substance abuse problems may use meaning-making to enhance their motherhood experiences and to better fulfill their maternal roles, especially when the surrounding discourses or norms usually position these women as “bad mothers.” The current study suggests these mothers have the potential to navigate the socio-cultural claims that they were “bad mothers” by appreciating their own commitment to maternal roles (Baker and Carson, 1999). Although some mothers may feel affected by the surrounding discourses and social standards of ideal motherhood, particularly when engaging in self-evaluations, the current findings suggest that it is possible for some to recognize their resilience in mothering and sufficient effort put into taking care of their children (Baker and Carson, 1999). Doing so may allow these mothers to have a higher level of self-efficacy and become more future-oriented when performing their childrearing activities. Furthermore, it is possible that their direct encounter with and care for their children are more important than cultural or societal pressures in enhancing their motivation to create meaning out of their situations and hence change their behavioral, emotional, or parenting outcomes (To et al., 2020). These findings are consistent with the findings of another qualitative study on disadvantaged Chinese mothers, which found the maternal identity was affected by the personal perception of the mother on mother-child relationships and interactions (To et al., 2020). Therefore, future research or services for these mothers may seek to emphasize the mother-child relationship when helping these individuals create meaning out of their situations and hence enhance their personal growth and parenting outcomes. Attention should also be paid to the socio-cultural contexts that these mother face, which may exert considerable influence on how they construct meanings of motherhood.

### Implications for Practice and Research

The current findings can provide insights into future initiatives aimed at enhancing the well-being and livelihood of mothers involved in problematic substance use and their children (Niccols et al., 2012). Meaning-making may be particularly relevant for these mothers as the rehabilitation process is often closely tied to other areas of the life of the recoveree, such as their maternal experiences, family relationships, and self-identity (Gunn and Samuels, 2020). Thus, future interventions should place greater attention on the narration of these mothers and reflection on their personal global and situational parental meanings, how these individuals cope with negative emotions stemming from the discrepancies between their global and situation meanings, and ways to enhance their meaning-making processes.

For clinical assessments, social service providers can broaden their conceptualization of the needs and challenges encountered by these mothers through understanding their meaning-making process. For interventions, the knowledge produced by this study can assist social workers and counselors in strengthening the meaning-focused coping of these mothers, as well as designing and implementing meaning-oriented parenting interventions with tailored content and goals (To et al., 2018). In addition to meaning-oriented interventions, practitioners can develop holistic and integrated programs in combination with other empirically supported parenting interventions to support these mothers and their families (Niccols et al., 2012). Furthermore, researchers and practitioners can also apply the meaning-making model to fathers with substance abuse problems to help address the unique needs of fathers.

While the current study marks a pioneering attempt to apply the meaning-making model in the context of mothers with substance use problems, several limitations should be noted. First, given the small sample size of the study, it remains unclear whether different demographic characteristics and backgrounds would influence the perceptions and experiences of mothers involved in substance abuse, especially in the context of meaning-making. Future studies involving a broader cross-section and greater diversity of participants can assist in expanding the current understanding of their needs. Second, longitudinal qualitative studies following mothers with substance abuse problems over time may be beneficial to analyzing the dynamic and evolving process of meaning-making throughout motherhood. Third, different data sources can be used to further supplement the understanding of parental meanings between mothers and fathers, and of between parents and their children. Fourth, there may be self-selection bias or social desirability processes at play given that an open recruitment process was used and that there were some common characteristics among the mothers (e.g., all participants had not abandoned their parenting responsibilities or relinquished care over their children). Future research should consider mothers who have chosen other options such as giving up their child for adoption. Likewise, although both field observations and in-depth interviews were conducted, it is difficult to fully examine the extent to which social desirability influenced the responses of participants. Finally, because this study recruited and interviewed participants with relatively positive parenting outcomes, more consideration should be given to the factors contributing to the emergence of negative outcomes in future research. Taken together, these limitations can provide the basis for future research directions.

Notwithstanding the limitations, this study provides a detailed description and analysis of the meaning-making process of mothers with substance abuse problems following childbirth, which contributes to the theoretical and practical advancement of knowledge and services for the maternal experiences, meaning-making, and change-making processes, and life and motherhood outcomes of this target group. Social service providers can also incorporate the findings from this study when supporting the needs of these mothers or when providing clinical assessments and interventions. Finally, the current findings can help to generate new service strategies to promote the well-being and outcomes of these mothers and their children.

## Data Availability Statement

The datasets presented in this article are not readily available because the data set is consisting of interview data, and confidentiality cannot be safeguarded. The data will therefore not be made available. Requests to access the datasets should be directed to siumingto@cuhk.edu.hk.

## Ethics Statement

The studies involving human participants were reviewed and approved by the Survey and Behavioral Research Ethics Committee of The Chinese University of Hong Kong (ref. number: 140021). The patients/participants provided their written informed consent to participate in this study.

## Author Contributions

S-mT initiated the project. He has been active in all phases of the project, including design, data collection, data analysis, and writing. Mw-Y has been active in data collection, data analysis, and writing. CL has been active in data analysis and writing. All authors contributed to the article and approved the submitted version.

## Funding

This study was funded by the Beat Drugs Fund Association of Hong Kong SAR Government (Ref. No. 140021).

## Conflict of Interest

The authors declare that the research was conducted in the absence of any commercial or financial relationships that could be construed as a potential conflict of interest.

## Publisher's Note

All claims expressed in this article are solely those of the authors and do not necessarily represent those of their affiliated organizations, or those of the publisher, the editors and the reviewers. Any product that may be evaluated in this article, or claim that may be made by its manufacturer, is not guaranteed or endorsed by the publisher.
